# APOEε4 and slow wave sleep in older adults

**DOI:** 10.1371/journal.pone.0191281

**Published:** 2018-01-25

**Authors:** Gregory J. Tranah, Kristine Yaffe, Caroline M. Nievergelt, Neeta Parimi, M. Maria Glymour, Kristine E. Ensrud, Jane A. Cauley, Sonia Ancoli-Israel, Sara Mariani, Susan Redline, Katie L. Stone, the Osteoporotic Fractures in Men Study (MrOS) Research Group

**Affiliations:** 1 Research Institute, California Pacific Medical Center, San Francisco, California, United States of America; 2 Departments of Psychiatry and Neurology, University of California San Francisco, San Francisco, California, United States of America; 3 Medical Center, San Francisco VA, San Francisco, California, United States of America; 4 Department of Epidemiology and Biostatistics University of California San Francisco, San Francisco, California, United States of America; 5 Department of Psychiatry, University of California San Diego, La Jolla, California, United States of America; 6 Division of Epidemiology and Community Health, University of Minnesota, Minneapolis, Minnesota, United States of America; 7 Center for Chronic Disease Outcomes Research, Minneapolis VA Medical Center, Minneapolis, Minnesota, United States of America; 8 Department of Medicine, University of Minnesota, Minneapolis, Minnesota, United States of America; 9 Department of Epidemiology, University of Pittsburgh, Pittsburgh, Pennsylvania, United States of America; 10 Department of Medicine, University of California San Diego, La Jolla, California, United States of America; 11 Division of Sleep & Circadian Disorders, Brigham & Women's Hospital, Harvard Medical School, Boston, Massachusetts, United States of America; 12 Departments of Medicine, Brigham and Women's Hospital and Beth Israel Deaconess Medical Center, Harvard Medical School, Boston, Massachusetts, United States of America; Nathan S Kline Institute, UNITED STATES

## Abstract

Slow wave (or stage N3) sleep has been linked to a variety of cognitive processes. However, the role of stage N3 in the elderly is debated. The link between stage N3 and episodic memory may be weakened or changed in the older adult population, possibly due to several altered mechanisms impacting the cellular structure of the brain. The bases for the age-related dissociation between stage N3 and cognition are not understood. Since *APOE*ε4 status is the strongest genetic risk factor for cognitive decline, we assessed whether the ε4 allele is associated with stage N3 sleep. Participants were from the population-based Osteoporotic Fractures in Men (MrOS) cohort with polysomnography and *APOE*ε4 genotype data (n = 2,302, 100% male, mean age 76.6 years). Sleep stages were objectively measured using overnight in-home polysomnography and central electroencephalogram data were used to score stage N3 sleep. Cognitive function was assessed using the Modified Mini Mental State Exam (3MS). The *APOE* rs429358 single nucleotide polymorphism, which defines the *APOE*ε4 allele, was genotyped using a custom genotyping array. Total time in stage N3 sleep was significantly higher (p<0.0001) among the 40 MrOS participants carrying two copies of the ε4 allele (62±5.2 minutes) compared with 43±1.5 minutes for carriers of one ε4 allele (n = 515) and 40±0.8 minutes for ε4 non-carriers (n = 1747). All results were independent of sleep efficiency, number of sleep cycles, and apnea hypopnea index. These findings support an association between *APOE*ε4 genotype and sleep stage N3 in the elderly. Increased total stage N3 duration among ε4/ε4 carriers does not appear to reflect compensation for prior cognitive decline and may reflect overactive downscaling of synapses during sleep. If confirmed, these results might in part explain the high risk of age-related cognitive decline and AD among APOE ε4/ε4 carriers.

## Introduction

Sleep disturbances and specific sleep disorders increase with advancing age [1–6] and elevate the risk for a variety of diseases and conditions [7–22] including cognitive impairment [23–33]. Recent research indicates that sleep may specifically impact the cellular structure of the brain, including the ability of the brain to clear toxic metabolites that accumulate with normal neuronal function [34]. This includes beta-amyloid [34], which accumulates in apolipoprotein E (*APOE*) ε4 genotype carriers [35] and contributes to synaptic degeneration and cognitive decline. While the *APOE*ε4 allele is a well-established risk factor for Alzheimer’s disease (AD) and age-related cognitive decline [36–42] as well as a risk factor for sleep apnea [43–51], *APOE*ε4 has not been assessed in relation to sleep stage distributions. To explore the hypothesis that *APOE*ε4 influences sleep architecture, we examined the effect of the *APOE*ε4 allele on sleep stage distribution measured in a large, population-based cohort of elderly participants with a focus on stage N3, which represents the synchronization of cortical EEG activity and has been implicated in memory formation in young populations [52–57]. Given the high risk of cognitive decline and AD among *APOE*ε4 carriers, identifying a genetic link with stage N3 sleep could advance the development of new approaches for interventions to slow the decline in cognitive function among ε4 allele carriers and that may also benefit the health and well-being of the elderly.

## Materials and methods

### Participants

Data were collected from participants of the Osteoporotic Fractures in Men (MrOS) Study which included participants who were at least 65 years old and excluded participants who required assistance with ambulation or had undergone bilateral hip replacement. In the MrOS study, 5994 older men were recruited from six geographical areas (Birmingham, AL; the Monongahela Valley near Pittsburgh, PA; Minnaepolis, MN; Palo Alto, CA; San Diego, CA; and Portland, OR) between 2000 and 2002. The MrOS Sleep Study, an ancillary study of the parent MrOS cohort, was conducted between December 2003 and March 2005 and recruited 3135 MrOS participants (68%) for a comprehensive sleep assessment and assessment of circadian gene polymorphisms. Of the 2,859 men who did not participate in this ancillary study, 344 died before the sleep visit, 36 had already stopped participating in the study, 332 were not invited because recruitment goals had already been met, 150 were not eligible and 1,997 refused. Among the participants of the MrOS Sleep Study, 2480 self-identified white men who had DNA extracted for genetic studies. Details regarding the studies have been published previously [58,59]. All data were collected with written informed consent as approved by the institutional review boards of the clinical sites (University of Alabama at Birmingham; University of Minnesota; University of Pittsburgh; Stanford University; Oregon Health & Science University, and University of California, San Diego) and the coordinating center (University of California, San Francisco). The Outcomes of Sleep Disorders in Older Men Study (MrOS Sleep Study) [60], an ancillary study of the parent MrOS Study, enrolled 3,135 participants from 2003–2005. Of the 3135 enrolled participants, 2911 had usable PSG data and, of these, 2745 (94%) with evaluable sleep staging data. The Modified Mini Mental State Exam [61,62] (3MS), an expanded 100 point version of the original MMSE, was administered in MrOS at the baseline and sleep exams (~3 years) [63]. The Pittsburgh Sleep Quality Index (PSQI) and Epworth Sleepiness Scale (ESS) questionnaires were administered at the sleep exam.

### Polysomnography

In-home, single night sleep studies using unattended polysomnography (PSG, Safiro, Compumedics, Inc., Melbourne, Australia) were performed as previously described [64,65]. The recording montage consisted of C_3_/A2 and C_4_/A_1_ electroencephalograms, bilateral electroculograms, a bipolar submental electromyogram, thoracic and abdominal respiratory inductance plethysmography, airflow (using nasal-oral thermocouple and nasal pressure cannula), finger pulse oximetry, electrocardiogram, body position (mercury switch sensor), and bilateral leg movements (piezoelectric sensors). Trained certified staff members performed home visits for setup of the sleep study units. After sensors were placed and calibrated, signal quality and impedance were checked, and sensors were repositioned as needed to improve signal quality, replacing electrodes if impedances were elevated, using approaches similar to those in the Sleep Health Heart Study [66]. After studies were downloaded, they were transferred to the Case Western Reserve University Reading Center (Cleveland, OH) for centralized scoring of sleep stages and sleep disorders by a trained technician using standard criteria [67,68]. Sleep was staged in 30-sec scoring epochs and electroencephalogram data were used to score stage N3 using the criteria of Rechtschaffen and Kales [67]. Total time (minutes) and percent time [100*(Number of minutes scored as stage 3–4)/(total sleep time)] in stage N3 were calculated for these analyses. The apnea hypopnea index (AHI) was calculated as the total number of apneas and hypopneas per hour of sleep (4% desaturation) [69]. Sleep cycles were defined using a modified version of the rules detailed by Feinberg and Floyd [70]. A cycle was defined as a period of at least 15 minutes of NREM sleep starting with stage N2 or N3 and terminated by the end of a period of REM sleep lasting at least 5 minutes for all cycles except cycle 1, or a period of wake or stage N1 lasting 15 minutes or longer. Two epochs of REM sleep separated by no more than 15 minutes of NREM sleep or wake were merged into a single REM period. Average absolute spectral power density in log_10_(uV^2/Hz) was calculated for each sleep cycle for the following bands: Slow oscillation: 0.25–1 Hz, Delta: 1.25–4 Hz, Theta: 4.25–8 Hz, Alpha: 8.25–12 Hz, Sigma: 12.25–15 Hz, and Beta: 15.25–20 Hz. There was excellent inter-scorer reliability for the key sleep parameters, including stage N3 (ICCs >0.92). Several additional sleep parameters were scored, including: wake after sleep onset time and sleep efficiency.

### APOE genotyping

The *APOE* rs429358 SNP, which defines the *APOE*ε4 allele, was genotyped using a custom Illumina Golden Gate assay (Illumina, San Diego, CA, USA) that included 529 SNPs as previously described [71]. All individuals in this study are of European ancestry and are unrelated. Of 2,745 MrOS participants with PSG, 2,302 were genotyped for the rs429358 C/T alleles, which define *APOE*ε4 status by the presence of the C allele. All participants in this analysis are of European ancestry.

### Statistical analyses

Associations between *APOE*ε4 status and sleep staging were based on 2,302 MrOS participants with both PSG and rs429358 genotype data. All PSG and cognition measures were treated as continuous variables in analyses examining both additive and dominant genetic models to examine *APOE*ε4 associations for total time and percent time spent in stage N3. All regression models were adjusted for age and clinic site with additional models including measures of sleep duration (TST), quality (WASO time and sleep efficiency) and AHI. Cognitive function change (~3 years) from the visit prior to sleep assessment was calculated and used to stratify stage N3-cognition analyses into high and low decline groups. All analyses were analyzed using SAS version 9.4 (SAS Institute Inc, Cary, NC).

## Results

Differences in age, cognition, sleep performance and stage N3 across three *APOE*ε4 categories are shown in Table 1. Total stage N3 time was significantly higher among 40 MrOS participants carrying two copies of the ε4 allele (62±5.2 minutes) compared with 43±1.5 minutes for carriers of one ε4 allele (n = 515) and 40±0.8 minutes for ε4 non-carriers (n = 1747). The *APOE*ε4 association with stage N3 time (p = 0.0002) was not altered by adjustment for 3MS (p = 0.0001). We examined the *APOE*ε4-stage N3 association among MrOS participants who had experienced either ≥2 point decline or <2 point decline (based on median change) from the previous 3MS assessment to the 3MS measured at the sleep visit. Total stage N3 time was significantly higher among participants carrying two copies of the ε4 allele when compared to the other genotype categories in both the group with ≥2 point 3MS decline (p<0.0001) and the group with <2 point decline (p = 0.0002) and the test for interaction between *APOE* allele and 3MS decline was not significant (p = 0.21). In addition, the APOEε4 association with 3MS (p<0.0001) was not altered by adjustment for stage N3 time (p<0.0001) and the test for interaction between APOEε4 and stage N3 time in the association with 3MS was statistically significant (p = 0.006). The APOE ε4/ε4 genotype was associated with higher sleep efficiency and lower WASO but not the PSQI or ESS assessments. Spectral power density of the C3-A2 EEG in the slow oscillation (0.25–1 Hz) band was calculated for the final sleep cycle and did not differ among *APOE* alleles (Table 1). The total number of sleep cycles did not differ among *APOE* alleles (Table 1). The *APOE* associations with total stage N3 time did not change after additional adjustment for sleep efficiency (ε4/ε4 = 59±5.2 minutes, p = 0.0003), wake after sleep onset (ε4/ε4 = 60±5.2 minutes, p = 0.0002), number of sleep cycles (ε4/ε4 = 64±5.6 minutes, p = 0.0001), or AHI (ε4/ε4 = 62±5.1 minutes, p<0.0001).

**Table 1:** Characteristics of MrOS participants with PSG sleep staging and *APOE* genotyping.

		*APOE*ε4 alleles			
	0	1	2	p-value^a^	p-value^b^
n (%)	1747 (75.8)	515 (22.5)	40 (1.7)		
	mean (SE)	mean (SE)	mean (SE)		
Age, y	76.6 (0.1)	76.5 (0.2)	75.6 (0.9)	0.48	0.13
3MS (0 to 100)*	93.4 (0.1)	92.4 (0.2)	90.2 (0.8)	< .0001	0.003
Change 3MS*^c^	-1.01 (0.1)	-1.47 (0.2)	-3.34 (0.7)	0.0025	0.0026
Time in stage N3 (min)*	40 (0.8)	43 (1.5)	62 (5.2)	< .0001	< .0001
Change 3MS ≥ -2	41 (1.0)	42 (1.8)	61 (7.4)	0.03	0.008
Change 3MS < -2	38 (1.4)	45 (2.5)	64 (7.5)	0.0005	0.002
Time in REM (min)*	70 (0.7)	68 (1.2)	65 (4.5)	0.21	0.13
Time in stage N1 (min)*	24 (0.3)	24 (0.6)	22 (2.1)	0.68	0.48
Sleep efficiency (%)*	76 (0.3)	76 (0.5)	81 (1.9)	0.05	0.01
Wake after sleep onset (min)	115 (1.6)	116 (2.8)	86 (10)	0.02	0.005
Apnea hypopnea index (4% desat)*	13 (0.3)	12 (0.6)	14 (2.1)	0.57	0.56
Number of sleep cycles*	4.7 (0.03)	4.6 (0.1)	4.6 (0.2)	0.50	0.97
Spectral power density- SOsc (log_10_(uV^2/Hz))*^d^	1.9 (0.01)	1.9 (0.02)	2.0 (0.6)	0.15	0.06
Arousal index	24 (0.3)	23 (0.5)	24 (1.9)	0.10	0.93
PSQI*	5.6 (0.1)	5.5 (0.1)	5.3 (0.5)	0.69	0.55
ESS*	6.1 (0.1)	6.1 (0.2)	5.7 (0.6)	0.76	0.47
Hypnotics, n	38	10	0		

*Adjusted for age

^a^ Linear p-value across three genotypes

^b^ P-value comparing carriers of two ε4 alleles to all others

^c^ Baseline to sleep visit ~ 3 years.

^d^ Spectral power density of the C3-A2 EEG in the slow oscillation (0.25–1 Hz) band for the final sleep cycle.

PSQI = Pittsburgh Sleep Quality Index; ESS = Epworth Sleepiness Scale

## Discussion

In the present study of >2,300 older men, we confirmed a significant association between *APOE*ε4 status and lower cognitive function scores and unexpectedly found that *APOE*ε4 was associated with increased stage N3 duration. Average total stage N3 time was >50% longer among elderly *APOE* ε4/ε4 carriers (those who at the highest risk of developing AD and age-related cognitive decline [36–42]) when compared to those with one or no copies of the ε4 allele. While those with the *APOE* ε4/ε4 genotype had more stage N3 time, stage N3 was not associated with cognitive decline in MrOS [72], even after adjustment for APOEε4 statusConsistent with increased stage N3 time among ε4/ε4 genotype carriers, the *APOE* ε4/ε4 genotype was also associated with higher sleep efficiency and decreased WASO, indicating overall less arousability. This finding was unexpected but could reflect a compensatory mechanism wherein the consolidation process is upregulated in reaction to declining cognition. In order to test this possibility we examined the *APOE*ε4-stage N3 association among *APOE* ε4/ε4 participants who had experienced either high or low ~3-year decline from the previous 3MS assessment. Total stage N3 time did not differ between participants who had experienced either high or low declines suggesting that the longer stage N3 time among ε4/ε4 carriers is not compensating for prior cognitive decline. It has been suggested that the negative correlations previously observed between stage N3 and subsequent learning in older adults is unlikely due to memory consolidation [73], and might be explained by another function of stage N3 such as synaptic downscaling or pruning [74]. During waking hours, encoding leads to increased synaptic weights which eventually results in the saturation of synaptic networks. The downscaling of certain synapses which takes place during stage N3 likely serves to ensure the maintenance of balanced synaptic input. The negative correlations observed between stage N3 and memory in older adults, however, may reflect an altered balance between daytime encoding and nighttime downscaling wherein older adults engage in less daytime encoding than younger adults [75] but over-downscale if they are still gaining relatively high amounts of stage N3. Thus, an age-related proportional increase in downscaling would presumably become detrimental to memory functioning if synapses that could otherwise help encode new memories are pruned. Such sleep-dependent synaptic downscaling has been observed in Drosophila [76,77].

Synaptic plasticity and scaling are crucial mechanisms in memory flexibility and AD onset [73,78,79] with experimental downscaling of AMPA receptors contributing directly to AD pathology [78]. In light of the genetic association data presented herein, it is possible that *APOE* ε4/ε4 carriers are experiencing “overactive downscaling” [73]. APOE is produced by the glia in the central nervous system [80], while APOE receptors are expressed on the neuronal cell surface where they mediate lipoprotein uptake and signal transduction [81,82]. It has been proposed that APOE isoforms may differentially accelerate synapse dysfunction and AD dementia by interfering with APOE receptor-dependent neuromodulation [81]. Of the three *APOE* isoforms (ε2, ε3, and ε4), *APOE*ε4 selectively impairs synaptic plasticity and NMDA receptor phosphorylation by Reelin, a modulator of synaptic strength [79]. This selective impairment of APOE receptor signaling by the APOEε4 isoform leads to a decrease in synaptic responsiveness [79]. The APOEε4 isoform also reduces glutamate receptor function and synaptic plasticity by selectively impairing APOE receptor recycling in primary neurons [79].

Sleep also impacts the cellular structure of the brain, including the ability of the brain to clear beta-amyloid, contributing to synaptic degeneration and cognitive decline [34]. For example, chronic sleep restriction increases amyloid-B plaque formation in the amyloid precursor protein transgenic mic [^34^]. Sleep also impacts the ability of the brain to remove potentially toxic biomolecules that accumulate with normal neuronal function, including beta-amyloid [34], which accumulates in *APOE*ε4 genotype carriers [35]. This "glymphatic system" is strongly stimulated by sleep and is associated with an increase in interstitial volume, possibly by shrinkage of astroglial cells, leading to clearance activity. Clearance during sleep is as much as two-fold faster than during waking hours [34]. Thus, glymphatic dysfunction may play a role in the pathogenesis of neurodegeneration as well as maintenance of cognition. However, it is also possible that accumulation of beta-amyloid and other toxic metabolites may influence synaptic connectivity, and thus influence neurophysiological mechanisms of sleep, altering sleep state distributions [83].

Few studies have examined the role of *APOE* genotype in both sleep and cognitive performance [84,85]. In a study of 126 cognitively normal adults (30–70 years), chronic daytime somnolence was associated with a distinctive decline in verbal memory among *APOE* ε4/ε4 carriers as compared to *APOE*ε4 heterozygotes and noncarriers [84]. A second study examined how sleep parameters in AD patients change over time as a function of *APOE*ε4 status [85]. Among forty-four community-dwelling adults with a diagnosis of probable AD there was greater deterioration on sleep parameters (increased WASO time and decreased TST and sleep efficiency) in non-*APOE*ε4 carriers when compared with positive *APOE*ε4 carriers [85]. Associations between sleep performance and cognition also differed between *APOE*ε4 carriers and non-carriers [85]. The *APOE*ε4 allele has also been identified in studies as a risk factor for sleep apnea, although the associations have not been consistent [43–51]. Despite these previous *APOE*ε4 -sleep associations, the *APOE*ε4-stage N3 results reported herein were independent of objective measures of sleep continuity as well as apnea hypopnea index.

In conclusion, these findings support a role for *APOE*ε4 genotype in the regulation of stage N3 in the elderly. Despite the established role of stage N3 in executive function [52,53] and memory consolidation [54–57] in younger and middle-aged adults, the link between stage N3 and cognitive function may be weakened or changed in older adult population [73,86–89] (including the MrOS study [72]). The negative correlations between stage N3 and subsequent learning in older adults could involve several altered mechanisms [74,90] (including memory consolidation, synaptic downscaling, or other macromolecular changes during stage N3 [83]). As demonstrated in previous studies, increased stage N3 might predicts worse episodic memory consolidation in the elderly [88] and may be detrimental to morning recall [73,91–94]. Similar changes to the sleep–memory link have also been reported for old rodents [95,96] showing that normal hippocampal reactivation is not preserved in older rats and that deep sleep was associated with worse memory performance as measured by performance on the Morris Water Maze task. Further research is needed to confirm the *APOE*ε4- stage N3 associations and to identify the mechanism(s) by which *APOE*ε4 alterations impact sleep architecture. Quantitative EEG analysis will be useful also to elucidate whether *APOE* influences homeostatic drive and sleep dynamics, and whether such changes associate with cognition. The important strengths of this study are the large number of participants with overnight PSG, longitudinal cognitive function measures, and *APOE*ε4 genotyping. An additional study strength is the use of a large cohort of older adults who were not selected on the basis of sleep or cognition characteristics. This study, however, was limited to white men and may not be generalizable to other populations or women. If it is confirmed that increased total stage N3 time among *APOE* ε4/ε4 carriers reflects an overactive downscaling of synapses during sleep, these results might in part explain the high risk of AD and age-related cognitive decline among *APOE* ε4/ε4 carriers.
